# Physical condition of Olyset® nets after five years of utilization in rural western Kenya

**DOI:** 10.1186/1475-2875-12-158

**Published:** 2013-05-10

**Authors:** Paola Mejía, Hailay D Teklehaimanot, Yihenew Tesfaye, Awash Teklehaimanot

**Affiliations:** 1 The Earth Institute, Columbia University, 475 Riverside Drive, Suite 401, New York, NY 10115, USA; 2 Center for National Health Development in Ethiopia, Columbia University. Bole Sub City, Kebele 06, H No 447, PO Box 664, code 1250, Addis Ababa, Ethiopia

**Keywords:** Long-lasting insecticidal nets, Insecticide-treated nets, LLINs, ITNs, Olyset, Durability, Kenya, Millennium Villages Project

## Abstract

**Background:**

Long-lasting insecticidal nets (LLINs) are a cornerstone of malaria control at present, and millions are used each day across the globe. However, there is limited information about the durability of LLINs under different conditions of utilization and there is no consensus about when a LLIN ceases to be protective due to physical deterioration. This knowledge is important for malaria control programmes to plan for procurement and replacement.

**Methods:**

A cross-sectional survey of 208 households where Olyset® nets distributed five years ago were still present was conducted in the village of Sauri, western Kenya, in the context of the Millennium Villages Project. Information on bed net utilization and maintenance was collected in each household through a structured questionnaire, and one five-year-old Olyset® net from each sampled household was randomly selected and collected for physical examination. All holes larger than 0.5 cm were measured in each net, registering their position, and a hole index was calculated following WHO guidelines. Nets were classified as in good condition, moderately damaged or badly torn based on the hole index. The analysis explored the associations between demographic and socioeconomic characteristics of households, patterns of bed net utilization and maintenance and physical condition of the nets. Additional analysis was conducted using malaria prevalence data collected in a separate survey to explore if there was any association between the condition of the net collected in a household and the presence of malaria parasites in members of that household.

**Results:**

81.4% of Olyset® nets distributed five years ago were still present in the surveyed households, and 98.97% of the nets were reportedly used the previous night. Nets had an average of 34.2 holes (95% CI 30.12-38.22), and the mean hole index was 849 (95% CI 711–986), IQR 174–1,135. 15.2% of nets were still in good condition, 46.1% were moderately damaged and 38.7% were badly torn after five years of utilization. There was no association between household characteristics or patterns of bed net utilization or maintenance and physical condition of the nets. The only predictor of the physical condition of the net was the cleanliness at the time of examination. There was a difference of 17.6 percentage points in the proportion of households with at least one blood smear positive for *Plasmodium falciparum* between households with a net in good condition (5.3%) and those with a moderately damaged or badly torn net (22.9%), 95% CI (0.04-0.305), t=2.77 with unequal variance, p=0.009.

**Conclusions:**

Olyset® nets were used extensively in Sauri, western Kenya after five years of distribution, regardless of their physical condition. However, only 15% were found in good condition. Nets in good condition seem to be still protective after five years of utilization, while nets with more than 100 cm^2^ of holed surface may be associated with higher malaria parasitaemia at household level. Continued replacement of damaged nets and promotion of net maintenance and repair may be necessary to maintain the protective effectiveness of LLINs.

## Background

Long-lasting insecticidal nets (LLINs) are a cornerstone of the malaria control strategy globally. Many countries have embarked in large-scale distribution of LLINs in the last few years, and WHO estimates that between 2008 and 2010, approximately 294 million of ITNs were delivered in sub-Saharan Africa, enough to cover 73% of the population at risk of malaria [1]. Most insecticide-treated nets delivered in Africa are LLINs. There are at present four LLINs that have received full recommendation from WHO: Olyset®, PermaNet®, Yorkool® LN, and Interceptor®, and another nine LLINs have received an interim recommendation [2]. These recommendations are based on the fulfilment of WHO efficacy criteria in laboratory and field trials, which are minimum standards assumed to confer the net at least three years of useful life. Regardless of millions of LLINs in use by households in rural Africa, there is at present very limited knowledge about their lifespan under normal field conditions. Information on the durability of LLINs is important for malaria control programmes to plan for procurement and replacement of nets.

The longevity of LLINs depends on multiple factors, which may include material (polyester, polypropylene or polyethylene), housing conditions, whether they are used with beds or mats, who sleeps under them, whether they are put away during the day, seasonality of use, weather, and length of time that bed nets have been in regular use in a particular area. Thus, the durability of LLINs will vary depending on the characteristics of the community using them, and no single product is the best choice for all circumstances.

Understanding the factors that influence LLIN durability will help malaria control programmes in selecting the most appropriate products for local conditions, and can help to tailor behaviour change communication campaigns to support better utilization and care of LLINs. WHO has defined LLIN durability as encompassing survivorship, fabric integrity and insecticidal activity (bioefficacy) and recently released guidelines for evaluating the durability of LLINs in field conditions [3].

Olyset® Net was the first LLIN to be fully recommended by WHO in 2001 [4]. Olyset® is made of single filament polyethylene (minimum 150 denier), blended with permethrin 2% as active ingredient, at a concentration of 1,000 mg permethrin per m^2^. The insecticide moves through the fibres by diffusion. Its mesh is 4 mm × 4 mm wide to allow for better ventilation in hot climates, and its sturdy fibre makes it resistant to harsh conditions [5].

The purpose of the present study was to evaluate the durability of Olyset® nets after five years of utilization in the village of Sauri, in western Kenya, which is part of the Millennium Villages Project. The Millennium Villages Project (MVP) was started in 2005 with the purpose of demonstrating that through the integrated delivery of scientifically proven interventions across multiple sectors, the Millennium Development Goals can be achieved even in the most challenging agro-ecological settings in rural Africa. Spanning 14 sites in 10 countries, MVP works with communities identifying problems and priorities and investing in health, education, agriculture, and infrastructure and business development [6,7]. The Millennium Village of Sauri (Sauri MV) was the first of such villages to become part of the project.

The present study evaluated the physical condition of five-year-old Olyset® nets and collected samples for bioefficacy studies, insecticidal content and textile strength analysis. This paper reports the physical condition of the nets and examines its association with household characteristics, patterns of utilization and maintenance, and also explores the association between bed net condition and malaria infection documented among household members in a previous malariometric survey conducted seven months earlier as part of the impact evaluation of the Millennium Villages Project.

## Methods

### Study site

**Figure 1:**
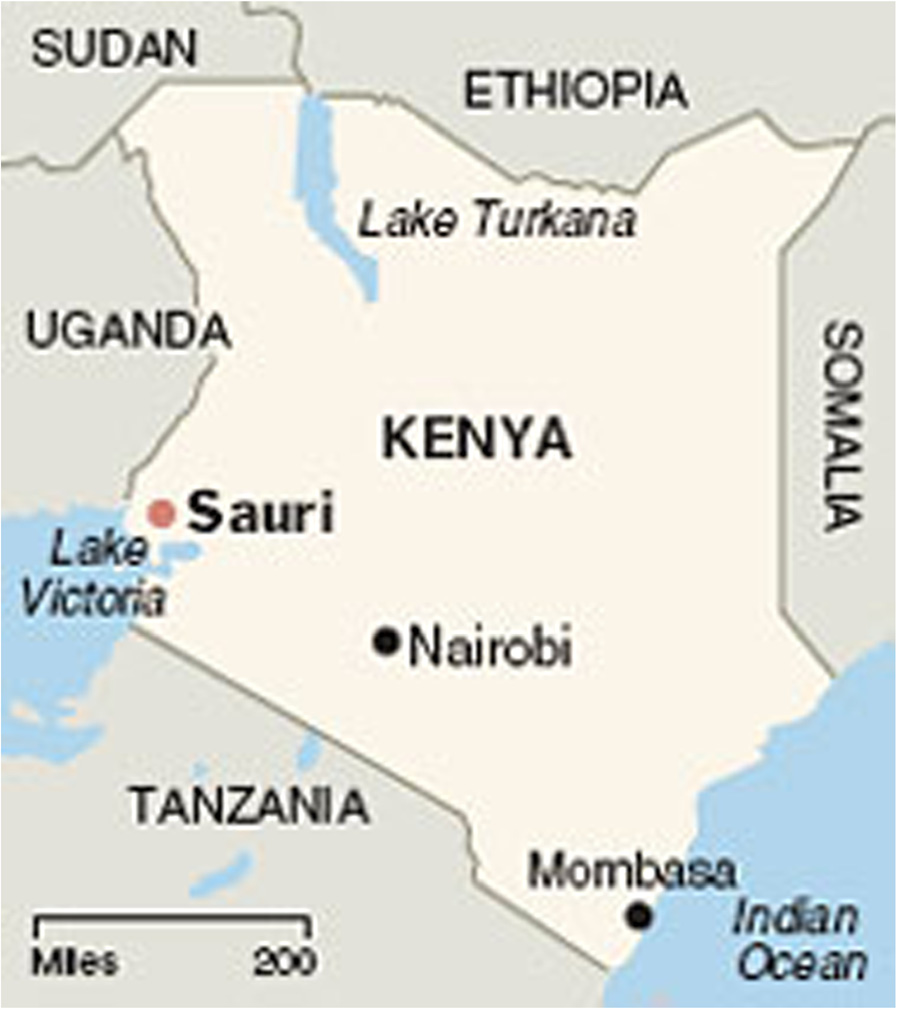
Map of Sauri Millennium Village.

Sauri is a Millennium Village in western Kenya, a community that is participating in the Millennium Villages Project of The Earth Institute, Columbia University. Sauri MV comprises 11 sub-villages with a population of 65,000 and is located in the Kenyan highlands in Yala Division, Siaya District, Nyanza Province, at 34.75° longitude east and 0.24° latitude north, 30 km north of Lake Victoria and 1,400-1,500 m above sea level (Figure 1). The average temperature in the area is 24°C, ranging from 18-27°C with an annual rainfall of 1,800 mm. Rainfall is bimodal, divided in a long rainy season from March to June and a short rainy season from September to December. Subsistence agriculture is the main livelihood in the area. *Anopheles funestus* and *Anopheles gambiae sensu stricto (s.s)* are the main anopheline species found in the study area, and the entomological inoculation rate (EIR) for the area was estimated to be seven infective bites per person per year in 2005. (Millennium Villages Project, unpublished data). That same year, malaria prevalence in children under five years of age was 55.8%, and 49% for all the population of Sauri MV at baseline. (Millennium Villages Project, unpublished data).

### Study design

In April/May 2005, as part of the health interventions of the MVP, 38,000 Olyset® nets were donated by its manufacturer, Sumitomo Chemical Co. Ltd (Japan), and distributed for free to all households in Sauri MV, at an average of two bed nets per household. In November 2010, preceding the distribution of new Olyset® nets for all households to replace the five-year-old nets, a cross-sectional study was conducted to collect and examine a sample of five-year-old nets still in use in Sauri MV’s households.

A registry of households that had received an Olyset® net in 2005 was obtained from MVP. The Sauri MV comprises 11 sub-villages, so a stratified random sample of 200 households was drawn from the registry, accounting for difference in sub-village size, with additional households sampled in the event that selected households no longer had an Olyset® net from the 2005 distribution. The sample size was chosen assuming that a five-year-old Olyset® net would be present in approximately 50% of households, within 0.07 percentage points with 95% confidence (n=196). In total, 278 households had to be visited in order to find 200 households that still owned at least one Olyset® net distributed five years before, since many households did no longer have any of the nets they had received in the initial distribution. The visits were made by 14 community health workers, who were known to the households because they regularly visit them to provide primary health care services such as home management of malaria, growth monitoring and immunization follow-up. The community health workers had participated as enumerators in previous MVP surveys and received a two-day training for the conduction of the Olyset® net assessment. The study procedures were approved by Columbia University IRB (Institutional Review Board) and the local Kenyan IRB.

Consent to participate in the study was obtained from the head of each household and all Olyset® nets present in the household were inspected to identify if they were distributed in 2005. The Olyset® nets distributed at that time were white and had a distinctive strong fabric label, different in shape from that of Olyset® nets distributed in subsequent years through the antenatal clinic, and had the number 2005 marked with pen ink. Most of the nets retained the 2005 mark, but in the few that did not, the fact that the net was distributed in 2005 was easy to corroborate by the characteristic label.

A structured interview was conducted in each household that had at least one 2005 Olyset® net, collecting demographic information and documenting the number of sleeping sites -whether permanent or temporary-, the sleepers who used them, all nets of any kind present in the house, their utilization, age and frequency of washing. One 2005 Olyset® net was randomly selected from all 2005 Olyset® nets in a given household by assigning a number to each net and drawing one number from a bag. The selected net was placed in an individual polyethylene bag and a voucher for collecting a new Olyset® net in the Sauri Health Centre the same day was given to the head of the household. All collected nets were taken to the Sauri Health Centre for examination.

Collected nets were of two different sizes: 130×180×150 cm and 100×180×150 cm. Five cube-shaped frames to fit these two sizes were constructed locally to mount the nets for examination. The frames were wrapped in black plastic sheeting to provide a contrasting background for easier examination. All holes larger than 0.5 cm diameter were measured, noting their position by measuring the distance from the bottom edge of the net to the centre of the hole, as per WHO guidelines [3]. Holes in the top panel were recorded separately. Conditions of seams were also noted, recording an opening larger than 1 cm as a seam failure. If the hole was caused by burns, it was marked as B, and animal chewing was noted by AC. Evidence of repairs to the net fabric such as knots, patches or stitches was also noted. In the case of sewn repairs, the length of the stitch was recorded. All this information was recorded in the respective household questionnaire.

### MVP malaria survey

As part of its impact evaluation plan, the MVP conducted baseline surveys of malaria prevalence (2005) and follow-up assessments at year 3 (2008) and 5 (2010) in 300 randomly selected households in Sauri MV, proportionally sampled from strata defined by sub-village, wealth category and gender of head of the household. At each assessment, malaria parasitaemia has been evaluated through thick and thin peripheral blood smears collected from a stratified sample of 600 children and adults. The year 5 survey was conducted between March 30^th^ and May 10^th^, 2010, around seven months before the collection of the five-year-old Olyset® nets. Results of blood smears collected from households from which a five-year old Olyset® net was also collected were identified. Due to confidentiality requirements of the individual blood smear data, blood smears were identified only by household number and could not be linked to a specific household member.

### Analysis plan

All the data were entered into a database at the Center for National Health Development in Ethiopia, Columbia University. Descriptive statistics were used to summarize household characteristics, as well as the location of holes. Holes were classified into four categories following WHO guidelines: 1) 0.5-2 cm; 2) 2–10 cm; 3) 10–25 cm; 4) >25 cm. A hole index (HI) was calculated for each net and a weight was assigned for each category of hole corresponding to the estimated holed areas, on the assumption that the hole sizes in each category are equal to the midpoint. These weights were 1, 23, 196 and 578 for each of the above hole categories respectively [3]. Nets were classified in three conditions by hole index (HI) adapting the system suggested by Kilian (RBM: Measurement of Net Durability in the Field: Current Recommended Methodology, presented in Lyon, February 2012): 1) good condition if HI <64 (less than 100 cm^2^ of holed area; 2) moderately damaged if 64 <HI <768; and 3) badly torn if HI >768 (more than 0.1 m^2^ of holed area).

A binary logit model was fitted with nets in good condition as dependent variable and cleanliness of the net (dirty net=1, clean or slightly dirty net=0) and dependency ratio as binary and continuous predictors respectively. These variables were included in the final model after assessing the effects of all variables with a p-value <0.1 in a *χ*^2^ test of association with the binary outcome “net in good condition”. The variables tested included gender of the head of the household, number of months without enough food to eat, dependency ratio (ratio of dependent household members over the number of adults in the household), occupation, number of nets owned, location and type of sleeping site, hanging method practiced, number of sleepers under the net, age group of sleepers (adult, young children or older children), frequency and method of washing, drying methods, and the cleanliness of the net.

The analysis also explored if there was any association between the physical condition of the net collected from a given household and the presence of malaria parasites in members of that household. Presence of a positive blood smear in at least one household member was modelled as binary outcome, and a *t*-test was done to assess any difference in proportion of positive households between those with a net in good condition and those with moderately damaged or badly torn nets.

## Results

208 five-year old Olyset® nets were collected from 208 households. From these, 4 were excluded from the analysis because they did not have the distinctive label of the nets distributed in 2005, with 204 nets kept for the analysis.

### Characteristics of the household

A total of 897 people were reported to live in the 204 study households, with a mean age of 26.3 years. 9.8% of the population was under five years of age (n=88), and 15.9% was less than 15 years old. 30.9% of households had at least one child less than five years of age. Household characteristics are presented in Table 1.

**Table 1:** Demographic characteristics of households and bed net ownership, maintenance and utilization in Sauri, 2010

		**n**	**%**			**n**	**%**
**Household size**	**1**	14	7	**Location of surveyed net**			
	**2**	18	9	Sleeping room		164	82
	**3**	23	11.5	Living room		22	11
	**4**	19	9.5	Kitchen		14	7
	**5**	21	10.5				
	**6**	35	17.5	**Does the sleeping site need to be set up every night?**			
	**7**	28	14	Yes		47	23.3
	**8**	17	8.5	No		155	76.7
	**9+**	21	12.5				
				**How is the bednet used?**			
**Number of nets in household**	**1**	43	23.5	Hanging freely over the bed		75	37.9
	**2**	70	34.3	Hanging over the bed and tucked under the mat		105	53
	**3**	49	24	Hanging freely over sleeping mat/mattress on the floor		10	5.1
	**4**	24	11.8	Tucked under sleeping mat/mattress on the floor		8	4
	**5+**	18	6.4				
				**How frequently is the net washed?**			
**Head of household gender**				Monthly		8	44
Female		90	46	Every 2-3 months		10	5.6
Male		106	54	Three times a year		41	22.8
				Twice a year		55	30.6
**Head of household age**				Once a year		42	23.3
Mean	58.4			Period longer than a year		24	13.3
**Head of household education**				**How is the net washed?**			
None		106	58.6	Cold water		177	98.3
Primary school		37	20	Warm water		1	0.6
Lower secondary school		33	18.2	Hot water		2	1.1
Upper secondary school or higher		5	2.8				
				**What soap is used to wash the net?**			
**Head of household occupation**				No soap		1	0.5
Farmer		154	79.4	Locally made soap		2	1.1
Salaried (professional, government, NGO)		8	4.1	Commercial bar soap		135	73.4
Casual farm labor (does not own land)		2	1	Commercial powder soap		46	25
Casual non-farm labor		7	3.6				
Self-employment in household or business		10	5.2	**Is net rubbed against rocks?**			
No occupation		13	6.7	Yes		10	5.7
				No		166	94.3
**Rooms in house with sleeping sites**	**1**	49	24				
	**2**	77	37.8	**Where was the net dried after washing?**			
	**3**	60	29.4	Outside under sun		120	64.9
	**4+**	18	8.9	Outside under shade		65	35.1
**Number of sleeping sites in household**	**1**	26	12.8	**How clean is the net?**			
	**2**	50	24.5	Clean		24	12.1
	**3**	46	22.5	Slightly dirty		115	57.8
	**4**	37	18.1	Very dirty		60	30.2
	**5**	32	15.7				
	**6+**	13	6.4	**At least one household member positive for*P. falciparum***			
				No		126	79.3
**How many people sleep under the net**	**1**	139	70.6		Yes	33	20.8
	**2**	43	21.8				
	**3**	11	5.6				
	**4**	4	2				

### Bed net ownership

Households had a mean of 2.46 mosquito nets per household, 95% CI (2.29-2.63), with a median of two nets per household. They reported receiving on average 2.5 Olyset® nets in the initial mass distribution campaign of MVP five years earlier. We did not find any household that did not have at least one LLIN. There were on average 3.23 sleeping sites per household and 2.25 rooms with sleeping sites in a house. 23.5% of households had only one net, 34.3% of households had two, 24% of households had three, and 18.1% of households had four or more nets. The mean number of sleepers under each net was 1.34, and the ratio of nets per person was 0.56 (see Table 1).

### Survivorship and attrition

From a total of 513 nets distributed in 2005, 92 were no longer present, resulting in a survivorship of 82%. Overall, 68.6% of households still had all the Olyset® nets they had received five years earlier, while 20.1% of households had discarded one net, 8.8% had discarded two nets and 2.5% had discarded three nets. Among the nets that were discarded, 59.8% were discarded because they had worn out, 30.4% due to burns, 4.3% were lost, 3.3% were taken away by a family member, 1.1% was discarded because it was too small and another 1.1% was destroyed by rodents.

### Utilization

81.6% of nets were located in a sleeping room, 11% were located in a living room and 7% were located in a kitchen; 93.9% were reportedly used year round every night, while 2.8% were used every night but only seasonally, and 3.3% were used only occasionally. Despite the presence of a Community Health Worker (CHW) programme, where CHWs visit households and educate on LLIN proper utilization, only 57% used their nets tucked under the mattress, as recommended, while 43% left the net floating freely.

The majority (98.97%) of nets were reportedly used the night preceding the survey. Most nets (70.6%) were used by only one sleeper, while 21.8% were used by two sleepers, 5.6% by three sleepers and 2% by four sleepers. Moreover, most nets were used exclusively by adults, whether sleeping alone (59.6%) or two adults sleeping together (11.1%). Children under five sleeping with either one or two adults made up 5.6% of the total nets, while another 5.6% of nets were used by children under five sleeping with one or more children, or with a combination of adults and children.

Among the 626 nets found in the surveyed households, 91.3% were Olyset®, 7.6% were other type of LLIN, while 1.1% were non-impregnated nets. 77% of sleeping sites reportedly had a bed net to protect them (72.4% were protected by a LLIN). 36.6% of all sleeping sites had to be set up every night, meaning that the bed net also had to be hung up every night over the site. Overall, 73% of sleeping sites had a bed net hanging over at the time of the survey, and 73.6% of people reportedly slept under a bed net the night before the survey.

**Figure 2:**
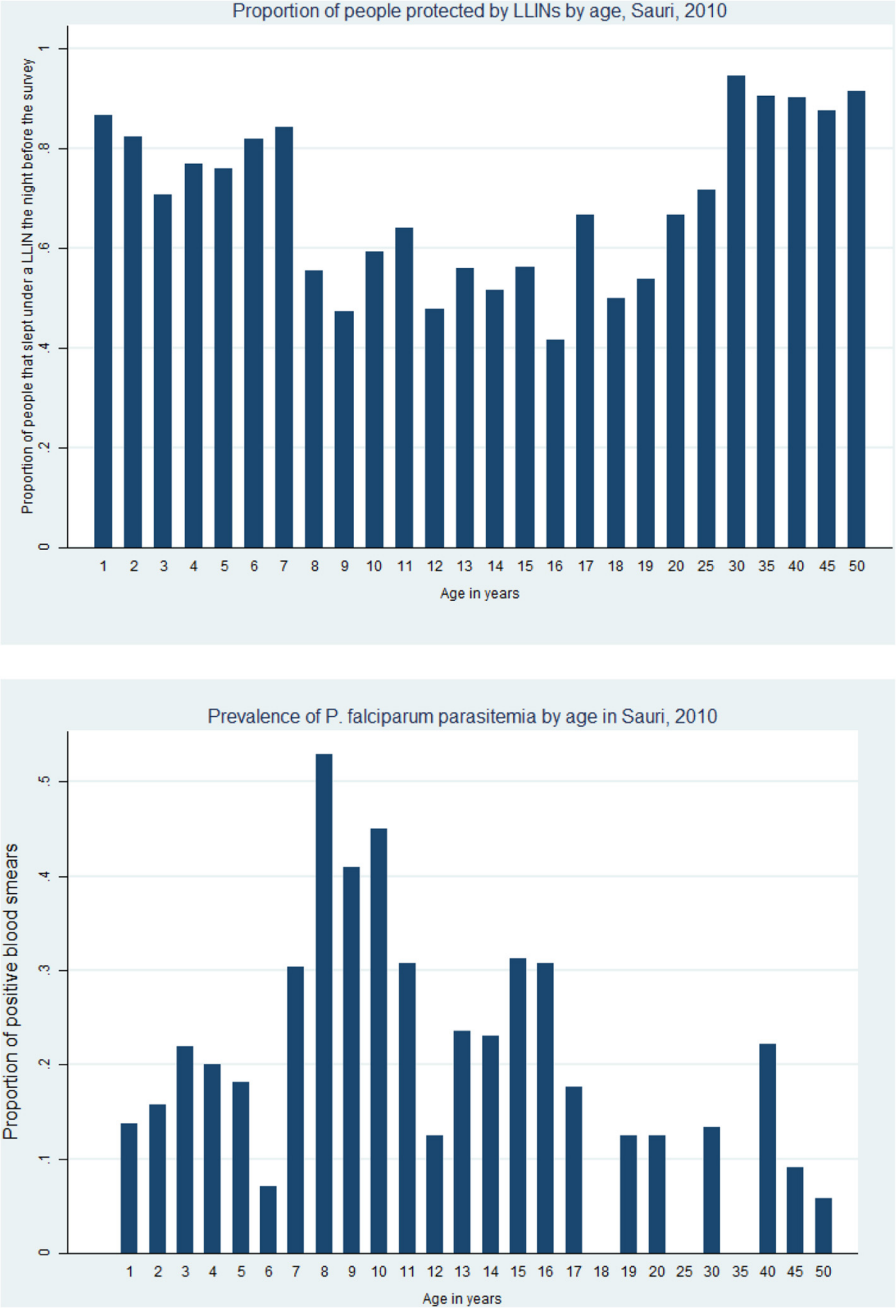
**Proportion of people sleeping under a LLIN by age, and proportion of blood smears positive for *P. falciparum*, Sauri, 2010.**

Children under five years of age and adults were more likely to sleep under a LLIN than older children, and protection with a bed net falls sharply around age eight, starting to increase again around 20 years of age. This contrasts with the age distribution of parasitaemia prevalence in Sauri for 2010, which is highest for children between 7 and 17 years of age, the same group more likely to sleep in an unprotected sleeping site (Figure 2).

Children between eight and 14 years of age had three times the odds of sleeping in an unprotected site than people of any other age. (OR=3.06, 95% CI (2.18-4.29). This finding is in line with what has been reported for sub-Saharan Africa in general, where school-aged children are the least protected by LLINs but represent the largest reservoir of infection [8].

### Care of bed nets

Of the 204 five-year old Olyset® nets, 16 had never been washed (7.8%). Most households wash their nets between one and three times a year (76.7%), with a mean of 10 washes since they received the net (95% CI 8.7-11.3). The vast majority wash their nets with cold water (98.3%), using commercial soap in either bar (73.3%) or powder (25%) formulation. Only 5.7% of household reported rubbing the net against rocks or stones when washing. 64.9% dry the net under the sun after washing it, while 35.1% dry them under the shade. Survey enumerators subjectively classified the collected nets as clean (12.1%), slightly dirty (57.8%) or very dirty (30.1%).

### Physical condition

**Figure 3:**
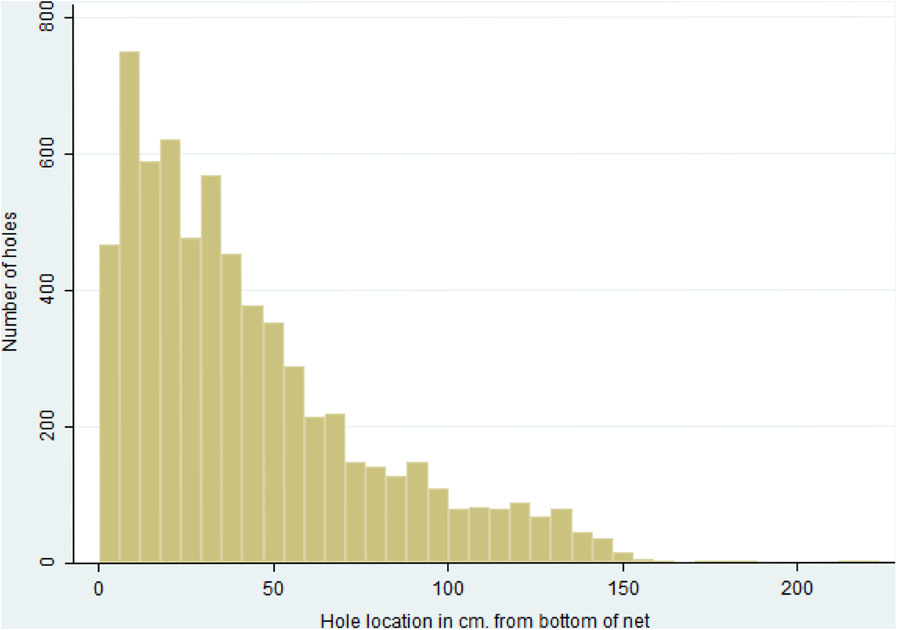
Distribution of the location of holes in five-year old Olyset® nets, measured from the bottom of the net.

Only one of the 204 nets had no holes. Very few had repairs: only four nets had stitches and three nets had knots to close large holes. 56.9% of holes were between 0.5 and 2 cm, and 75% of all holes were smaller than 3.3 cm. The mean hole size was 3.8 cm. Half of the holes in all nets were located below 34 cm, in the bottom quarter of the net (height is 150 cm), and 68% of holes were located in the lower third of the net. Only 2.3% of holes were located on the top panel of the net (Figure 3). The mean number of holes per net was 34.2 (95% CI 30.12-38.22), with a standard deviation of 28.5. The median number of holes was 27, ranging from 0 to 136.

The distribution of the hole index was right-skewed, with mean hole index 861 (95% CI 727–995.5), IQR 986 and median 571. Based on hole index, 15.2% of nets were found in good condition, 46.1% were moderately damaged and 38.7% were badly torn after five years of utilization.

### Factors determining physical condition of nets

In a univariate analysis with chi-square test, the only factors that were associated with having a net in good condition at a 5% level of significance were cleanliness of the net (whether the net was clean, slightly dirty or very dirty: Pearson *χ*^2^=7.58, p=0.023) and occupation (farmer, salaried or other: Pearson *χ*^2^=8.29, p=0.016) (Table 2). There was a statistically significant positive linear correlation between number of months of food insecurity and the hole index of the net (p=0.011), although it was relatively weak (Pearson correlation coefficient=0.18). There was also a negative association between a net in good condition (HI<64) and the number of sleepers sleeping under the bed net (Cochran Armitage trend test Z=1.977, two-sided p-value=0.048). In the final binary logit model, cleanliness was associated with having a net in good condition when dependency ratio and the age group of sleepers under a bed net were included in the model. (Wald *χ*^2^ test=4.4956, p-value= 0.0340). The OR of having a net in good condition was 2.924 times higher if the net was clean compared to dirty (OR 95% CI (1.085, 7.886)).

**Table 2:** Univariate analysis of factors associated with having a 5-year-old Olyset® net in good condition

**Factors**	**No of mosquito nets**	**Proportion of nets in good condition within category**	**OR (95% CI)**	**p-value**
**Gender of head of household**				0.38
Male	106	13.2	1.0	
Female	90	17.8	1.4 (0.7-3.1)	
**Education of head of household**				0.36
None	106	14.2	1.0	
Primary school	37	24.3	1.95 (0.8 – 4.9)	
Lower secondary school	33	15.2	1.1 (0.4 – 3.2)	
Upper secondary school	15	6.7	0.4 (0.05 – 3.5)	
**Occupation**				0.02**
Farmer	154	13	1.0	
Other	32	31.3	3.05 (1.3 – 7.4)	
Salaried	8	0	-	
**Number of nets owned by household**				0.9
1 to 2	118	16.1	1.0	
3 to 4	73	13.7	0.83 (0.36 – 1.89)	
5 or more	13	15.4	0.95 (0.19 – 4.62)	
**Location of sleeping site**				0.61
Sleeping room	164	15.2	1.0	
Living room	22	18.2	1.24 (0.39 – 3.95)	
Kitchen	14	7.1	0.43 (0.05 – 3.42)	
**Sleeping site has to be set up every night**				0.22
Yes	47	21.3	1.0	
No	153	13.7	0.59 (0.25 – 1.4)	
**Bednet tucked under bed at night**				0.11
No	85	10.6	1.0	
Yes	113	18.6	1.9 (0.83 – 4.45)	
**Number of sleepers under the net**				0.22
1	139	16.6	1.0	
2	43	9.3	0.52 (0.17 – 1.6)	
3	11	0	-	
4	4	0	-	
**Frequency of washing**				0.09*
Every month	8	37.5	1.0	
Every 2-3 months	10	0	-	
Three times per year	41	14.6	0.29 (0.05 – 1.5)	
Twice a year	55	18.2	0.37 (0.08 – 1.8)	
Every year	42	4.8	0.08 (0.01 – 0.62)	
Less than once per year	24	20.8	0.43 (0.08 – 2.5)	
**Rubbed against rocks when washing**				0.65
No	166	15.1	1.0	
Yes	10	10	0.63 (0.07 – 5.2)	
**Nets dried under the sun**				0.27
No	65	18.5	1.0	
Yes	120	12.5	0.63 (0.3 – 1.4)	
**Cleanliness of the net**				0.04**
Clean	24	33.3	1.0	
Slightly dirty	115	13.9	0.32 (0.12 – 0.88)	
Very dirty	60	10	0.22 (0.07 – 0.74)	

*p<0.1 **p<0.05.

### Malaria parasitaemia and condition of Olyset® nets

Blood smears were available from 152 households from which an Olyset® net was collected, and in some households more than one blood smear was available. A household was classified as positive for malaria if one or more blood smears were positive for *P. falciparum*.

A *t*-test was conducted to assess the difference in proportion of positive households between those where the collected net was in good condition vs. households with nets either moderately damaged or badly torn. There was a 17.6 percentage points difference between the two groups, with 22.86% of household positive for parasites among moderately damaged or badly torn nets and 5.26% of parasitemic households among those with nets in good condition (95% CI 0.04 – 0.3047), *t*=2.77=0.0087, with Satterthwaite unequal variances estimation (Probability of equal variances p=0.0045).

## Discussion

In the face of the millions of LLINs presently in use across the globe, knowledge about their physical resistance in different contexts and their performance over time is surprisingly limited. This paper reports the physical condition (fabric integrity) of Olyset® nets used in a rural area of western Kenya for five years.

A number of studies have assessed the durability of Olyset® nets under different conditions of utilization. A review of Olyset® nets conducted by WHOPES in 2009 presented the available information on the physical status of Olyset® nets of different ages (three and five years) in different countries. Information on five-year-old nets was available for five sub-Saharan African countries, and the mean number of holes per net (30 nets per study) was 12 in Burundi, 12.9 in Burkina Faso, 25.2 in Côte d’Ivoire, 33.8 in Burundi and 34.7 in Cameroon [5]. The present survey found a mean of 34.2 holes per net in rural western Kenya, second only to Cameroon. In contrast, a survey of Olyset® nets after five years of utilization in rural Laos found that 54.5% of nets had no holes, while only one net in the present study had no holes [9]. In a recent survey in south coastal Kenya, 78% of nets had holes, although they were of different ages and materials. However, the physical condition of nets in different environments (coastal plain, coastal slope, estuary, semi-arid inland) did not correspond with their age, with bed nets in the estuarine environment faring worst than the rest [10]. Sauri is located in a lowland, humid environment, and utilization of bed nets is very high, which may help to explain the relatively bad physical condition of the nets. Other studies have found more rapid deterioration in lowlands than in highlands, possibly due to seasonal utilization in highlands [11].

Regardless of their physical condition, nets continued to be used in Sauri and retention after five years was high, contrasting with the findings in the coastal region of Kenya suggesting that net owners discard damaged nets after 1.5 years of use [10].

Tami *et al.* followed up Olyset® nets that had been distributed seven years earlier in two Tanzanian villages and found that 100 out of 103 households surveyed still had these nets, and 95% of individuals still used them for mosquito protection [12]. The condition of 100 of these Olyset® nets was assessed, with 55% of them having 6 to 15 holes larger than 2 cm of diameter, with a mean of 13, ranging from 0 to 70. The present study found a similarly high rate of utilization of the existing Olyset nets (98.97%), although the attrition rate was higher (18.6%) and the physical condition after five years was poorer than that reported in Tanzania after seven years [13]. Since utilization is high regardless of the number of holes, it is important to understand how protective these damaged nets are, and further research is needed to define when a net ceases to prevent mosquito bites.

Studies have used different classifications of the condition of nets, from considering those in good condition with no holes, or intact, to defining different types of holes [14-18]. A consensus has emerged on the classification of the physical condition of ITNs, and recently WHO released guidelines for the monitoring of the durability of LLINs [3]. Those were the ones applied in this study.

The assessment of Olyset nets used in Sauri’s households for five years found a mean hole index of 849 (95% CI 711–986), IQR 174–1135. The only study so far that uses the WHO hole index is a recent publication on the effectiveness and durability of Interceptor LN in central India, which found a hole index of 252.2 after three years of utilization, and an average of 7.3 holes per net. However, only 27.2% of the surveyed population reported sleeping under the net every night year-round, with seasonal use more prevalent than regular, year-round utilization. The attrition rate of the Interceptor nets after three years was 18.1%, similar to the 18.6% attrition rate found for Olyset® nets after five years [19]. An evaluation of Interceptor polyester nets in western Uganda after three and a half years of utilization found 29% of nets in good condition, that is, with less than 100 cm^2^ of holed surface, which is the same parameter used in this study, which found only 15.2% of nets in good condition. However, attrition rate after three and a half years was 20.1%, higher than the 18.6% found in Sauri after five years, suggesting that a larger proportion of polyester nets are discarded due to damage [18].

A few longitudinal surveys have looked at the rate of physical deterioration of polyester nets [16,17]. However, evidence is scarce in comparing directly different types of LLINs under operational conditions. To our knowledge, the only study to date comparing different types of nets is a recent survey of the physical condition of three different types of LLINs after one year of utilization in a semi-arid environment in Chad. The study used a modified version of the WHO index, and found that 69.5% of nets were in poor or very poor condition, and that polyester nets had 4.22 times the odds of being in poor condition than Olyset® nets [20]. The survey of Olyset® nets after five years of utilization conducted in rural Laos found that among polyester nets found in the same households and of comparable age, 53.9% had at least one large hole (a hole that could be penetrated with a finger), compared to 18.2% of Olyset® nets [9].

The association between cleanliness of the nets and good physical condition suggests that a clean net may be a marker of a well-cared, well maintained net, and that Olyset® nets tolerate very well regular washing.

There is not a clear understanding of the relationship between protection, physical integrity and insecticide concentration. However, there is evidence that insecticide-impregnated bed nets inhibit blood feeding even when they are not physically intact, partly due to the repellent and irritant effect of pyrethroids [21]. This study used malaria prevalence data from households from which the five-year-old Olyset® nets were collected as a proxy for protection conferred by the nets. Malaria prevalence information was available only at the household level, not at the individual level, and thus the malaria blood smears do not necessarily correspond to individuals sleeping under the net sampled in a household.

There was a difference in malaria prevalence between households where a net in good condition was collected (5.26%) compared to those where the net was moderately damaged or badly torn (22.86%), for a 17.6% percentage points difference between good condition vs. moderately damaged or badly torn, 95% CI (0.04 – 0.30), p=0.0045. There was no difference in malaria prevalence between households with nets moderately damaged vs. badly torn. This appears to suggest that in order to be sufficiently protective, nets may need to be relatively intact. However, these results should be viewed with caution since we cannot identify the specific net under which an infected individual was sleeping, and there could be considerable variation in the physical condition of different nets in the same household. Moreover, physical condition and malaria infection were assessed at different times. Age of individuals sleeping under damaged bed nets and their age-related likelihood of malaria infection may also confound this association, although we did not find an association between physical condition of the net and whether it was used by adults or children.

A cross-sectional study in Laos examined the relationship between reported malaria episodes in households in the previous year and the compliance with the maintenance instructions of Olyset® nets. After two to three years of utilization, 39.3% of nets had holes, although the mean surface of holed area did not exceed 109 cm^2^, suggesting that most nets would be classified as in good condition under the present study, and the mean number of holes was 2.3. That at study did not analyse the relationship between the physical conditions of the nets and the reported malaria episodes, but found a relationship between reduced number of malaria episodes and appropriate maintenance behaviour of the nets [22]. The self-reported nature of the malaria cases in that study however is of very low specificity.

The present study also found that nets are rarely repaired, and that in spite of the presence of community health workers that educate households on bed net use and maintenance, roughly a quarter of the people are not sleeping under bed nets, and these sleepers are more likely to be children between 8 and 15 years old, the same age group with the highest malaria prevalence. In addition, 43% of bed net users do not use their net properly, leaving it hanging freely, which allows space for mosquitoes to enter. There is a need to reinforce behaviour change communication programs to encourage bed net utilization at all ages, proper hanging and maintenance and repair.

## Conclusions

Five years after the free distribution of Olyset® nets in the Millennium Village of Sauri in western Kenya, 82% of nets were still present in households, and 98.9% of those nets were reportedly used the night before the survey, even though only 15% of the nets were deemed in good physical condition. Nets in good condition were associated with lower malaria prevalence at a household level, suggesting that they were still protective after five years of utilization. Use of LLINs is lowest among older children, and there is a need for promotion of bed net utilization among school-aged children and improved communication of proper hanging and maintenance.

## Competing interests

Olyset® is a trademark of Sumitomo Chemical Company Ltd. Sumitomo Chemical Company is a partner of the Millennium Villages Project and has donated Olyset® Nets to cover all households in the Millennium Villages Project. It had no role in the design, conduction or conclusions of this study. There is no relationship, financial or otherwise, between the authors of this study and Sumitomo Chemical.

## Authors’ contributions

PM participated in the design of the study, collection of data, statistical analysis and drafted the manuscript. HT participated in the design of the study, collection of data and statistical analysis and helped draft the manuscript. YT participated in the collection of data and contributed to the preliminary analysis and drafting of the manuscript. AT conceived the study and participated in the design and analysis. All authors revised and approved the final manuscript.
